# Impact of weight maintenance and loss on diabetes risk and burden: a population-based study in 33,184 participants

**DOI:** 10.1186/s12889-017-4081-6

**Published:** 2017-02-06

**Authors:** Adina L. Feldman, Simon J. Griffin, Amy L. Ahern, Grainne H. Long, Lars Weinehall, Eva Fhärm, Margareta Norberg, Patrik Wennberg

**Affiliations:** 1 MRC Epidemiology Unit, Institute of Metabolic Science, University of Cambridge, Box 285, Cambridge Biomedical Campus, Cambridge, CB2 0QQ UK; 2 Department of Public Health and Clinical Medicine, Umeå University, 901 87 Umeå, Sweden

**Keywords:** Body mass index, Body weight change, Diabetes mellitus, Public health, Epidemiology

## Abstract

**Background:**

Weight loss in individuals at high risk of diabetes is an effective prevention method and a major component of the currently prevailing diabetes prevention strategies. The aim of the present study was to investigate the public health potential for diabetes prevention of weight maintenance or moderate weight loss on a population level in an observational cohort with repeated measurements of weight and diabetes status.

**Methods:**

Height, weight and diabetes status were objectively measured at baseline and 10 year follow-up in a population-based cohort of 33,184 participants aged 30–60 years between 1990 and 2013 in Västerbotten County, Sweden. The association between risk of incident diabetes and change in BMI or relative weight was modelled using multivariate logistic regression. Population attributable fractions (PAF) were used to assess population impact of shift in weight.

**Results:**

Mean (SD) BMI at baseline was 25.0 (3.6) kg/m^2^. Increase in relative weight between baseline and follow-up was linearly associated with incident diabetes risk, odds ratio (OR) 1.05 (95% confidence interval (CI) 1.04–1.06) per 1% change in weight. Compared to weight maintenance (±1.0 kg/m^2^), weight gain of > +1.0 kg/m^2^ was associated with an increased risk of incident diabetes, OR 1.52 (95% CI 1.32, 1.74), representing a PAF of 21.9% (95% CI 15.8, 27.6%). For moderate weight loss (−1.0 to −2.0 kg/m^2^) the OR was 0.72 (95% CI 0.52, 0.99).

**Conclusions:**

Weight maintenance in adulthood is strongly associated with reduced incident diabetes risk and there is considerable potential for diabetes prevention in promoting this as a whole population strategy.

**Electronic supplementary material:**

The online version of this article (doi:10.1186/s12889-017-4081-6) contains supplementary material, which is available to authorized users.

## Background

Since the positive outcome of several large scale trials demonstrated that lifestyle interventions among individuals at high risk for diabetes are effective for reducing disease incidence [1] the high-risk strategy has been the policy paradigm for prevention of diabetes [2, 3]. In many of the successful trials, weight reduction was a key component of the intervention [4, 5]. Indeed, the association between excess weight defined as overweight (BMI ≥25.0 kg/m^2^) and obesity (BMI ≥30.0 kg/m^2^), and increased incident diabetes risk is well established [6]. The association between BMI and incident diabetes appears to be approximately linear [7], making BMI a suitable candidate target for a population shift strategy, as proposed by Rose [8]. However, it is unknown how big such a shift would need to be to affect a substantial impact on the occurrence of diabetes in the population.

The potential public health impact of targeting a specific risk factor in the population is usually assessed by estimating the population attributable fraction (PAF), which is the proportion of cases that could be prevented if the risk factor, such as excess weight or weight gain, was eliminated. With regards to body weight and diabetes, most studies have focused on the potential impact on diabetes occurrence of eliminating excess weight (BMI ≥25.0 kg/m^2^) in the population. There has been considerable variability in the methods used to estimate these PAFs [9] which have ranged from 3% in women in the Framingham study [10], to 77% in a Finnish population [11]. However, as the probability that obese adults will attain normal weight is exceedingly low [12], a more realistic approach may be to study the potential population impact of achievable reductions in body weight, or of preventing weight gain [13, 14]. There is considerable evidence concerning the association between weight gain at a population level and diabetes risk [15], but there is much less evidence concerning the risk and public health impact associated with moderate weight loss or primary weight maintenance (*i.e.,* prevention of weight gain in adulthood). In addition, compared to the evidence for individual high-risk approaches, there is a dearth of evidence concerning population-based approaches for diabetes prevention. We aimed to investigate the public health potential of a whole population strategy for diabetes prevention by quantifying the impact on risk and population burden of diabetes of shifting the population distribution of body weight. To this end we used data from a large cohort study with objective longitudinal measures of weight and diabetes status.

## Methods

### Study population

The Västerbotten Intervention Programme (VIP) was initially established in 1985 as a community and individual-level programme to reduce the morbidity and mortality from cardiovascular disease in Northern Sweden [16]. The programme has evolved with the development of new national and international guidelines and methods, but the basics have remained the same. Participants are invited to their primary care centre during the year of their 30^th^ (until about 1995), 40^th^, 50^th^, and finally 60^th^ birthday for standardised health examinations including oral glucose tolerance tests (OGTT). For individuals found to have a BMI ≥30 kg/m^2^ the recommendation is that the attending health care practitioners provide counselling concerning lifestyle changes aimed at risk factor reduction, and those found to have Impaired Glucose Tolerance (IGT) are referred for a follow-up visit with a nurse, generally every second year (as with those found to have Impaired Fasting Glycaemia (IFG) after the concept was introduced in 2003) [16]. At every VIP visit the participants are asked to complete a comprehensive questionnaire that covers among other things lifestyle behaviour, health, and psychosocial status. Baseline participation rate over the study period has ranged from 48 to 67% of eligible people [16].

The data collected in VIP was used to conduct an observational prospective cohort study. All individuals first included in VIP between 1990 and 2003 at age 30, 40 or 50 were eligible to be included in the study population (*n* = 52,889). After excluding those who had prevalent diabetes at baseline (*n* = 1,280), missing baseline OGTT (*n* = 433) or who did not participate in the 10-year follow-up (*n* = 14,980), 36,196 participants with follow-up remained (70.7% of baseline participants). As diabetes patients are likely to lose weight following a diagnosis [17], we excluded participants who self-reported diabetes at follow-up (*n* = 487) to limit potential bias due to reverse causation. We also excluded all participants who at follow-up had missing OGTT data (*n* = 29). Finally, we excluded participants with missing, incomplete or erroneous data on weight and/or height (*n* = 1073, see details below) or missing data on any co-variate (*n* = 1,423), leaving a final study population of 33,184 participants with complete data available for analysis (Additional file 1: Figure S1). Written informed consent was obtained from VIP participants and ethical approval was granted by the Regional Ethical Review Board, Umeå University [Dnr 08–131 M].

### Assessment of diabetes

The outcome was newly detected diabetes at 10 year follow-up based on OGTT with a 75 g oral glucose load and measurements of fasting and 2-h capillary plasma glucose. Diabetes was defined as having fasting glucose ≥7.0 mmol/L or 2-h glucose of ≥12.2 mmol/L [18, 19]. For descriptive purposes we also defined IGT: fasting glucose <7.0 mmol/L and 2-h glucose ≥8.9 - < 12.2 mmol/L, and IFG: fasting glucose ≥6.1 - <7.0 mmol/L and 2-h glucose <8.9 mmol/L.

### Assessment of anthropometric data

Height and weight were measured in light clothing at the health examination at baseline and 10 year follow-up, and BMI was calculated as weight in kilograms (kg) divided by height in metres (m) squared. Except for small diurnal variation, people aged 30–50 years do not normally increase in height over 10 years follow-up, but some decrease in height may be expected [20]. To avoid unreasonable variation in BMI due to height measurement error or data entry error, we excluded all participants with larger than expected variation in height between baseline and follow-up (height gain >2 centimetres (cm) or height loss >2, >3 or >4 cm for those aged 30, 40 and 50 years, respectively). In addition, we excluded participants with BMI <10.0 kg/m^2^, or with an increase or decrease in BMI of >20.0 kg/m^2^ between baseline and follow-up. In total we excluded 1073 participants due to missing or erroneous height and/or weight data (see above).

Relative weight change was calculated as percent change relative to baseline weight *(((Weight*_*10year*_ *- Weight*_*baseline*_ *)/Weight*_*baseline*_ )*100). Absolute BMI change was calculated as *(BMI*_*10year*_ *- BMI*_*baseline*_ *)* in Δkg/m^2^. Weight maintenance between baseline and follow-up was defined as relative weight change ±3.0% of baseline weight [21], or as BMI change ±1.0 kg/m^2^.

### Assessment of other variables

Tobacco use was dichotomised as tobacco use (smoking or use of moist snuff,) or no current tobacco use. Marital status was dichotomised as married/cohabiting or single/divorced/widowed. Family history of diabetes was defined as presence of diabetes in any parent or sibling. Educational level was categorised as primary (mandatory only), any secondary or any tertiary. These variables were self-reported in the baseline VIP questionnaire.

### Statistical analysis

The association between the outcome, incident diabetes at 10 year follow-up, and within-individual change in weight and BMI was assessed using logistic regression generating odds ratios (OR) and 95% confidence intervals (CI). Four models were used to analyse the data: 1) BMI at 10 year follow-up was modelled as a categorical variable (<25.0; 25.0–29.9; 30.0–34.9; ≥35.0 kg/m^2^) stratified by baseline BMI category, with staying in the same category as the reference; 2) Absolute BMI change between baseline and 10 year follow-up was modelled as a continuous variable stratified by baseline age and BMI category; 3) Relative weight change between baseline and 10 year follow-up was modelled as a categorical variable (> +7.0%; +3.0 to +7.0%; ±3.0%; −3.0 to −7.0%; > −7.0%) stratified by baseline BMI category, with weight maintenance as the reference.; 4) Relative weight change between baseline and 10 year follow-up was modelled as a continuous variable, overall and stratified by baseline BMI category. Weight change categories were based on an established definition for weight maintenance [21], and gain or loss of 5 ± 2%. Stratification was achieved by including an interaction term between baseline BMI category and the exposure. Linearity in models 2 and 4 was tested by introducing a quadratic term. All models were adjusted for BMI at baseline (continuous) and the co-variates known to be associated with body weight and diabetes; sex, age at baseline (30, 40 or 50 years), calendar year at baseline (continuous), educational level, marital status, family history of diabetes and tobacco use. As a sensitivity analysis we excluded participants who were classified as underweight (<18.5 kg/m^2^) at baseline. To assess the impact of weight or BMI change on the cumulative 10-year incidence of diabetes in the population we estimated PAFs with 95% CI using the “punafcc” command in Stata. For calculating PAFs the modelled counterfactuals were: weight maintenance vs. any gain (i), moderate weight loss vs. maintenance or any gain (ii), and large weight loss vs. moderate loss or maintenance or any gain (iii). PAFs were calculated using the formula (pd*((OR-1)/OR)) [22] where *pd* is the proportion of incident diabetes cases with the exposure (*e.g.,* those with any weight gain in (i)) and OR is assumed to be a suitable approximation of the risk ratio (RR) due to the low prevalence of the outcome [23]. All data were analysed using Stata v. 13 for Windows.

## Results

**Fig. 1:**
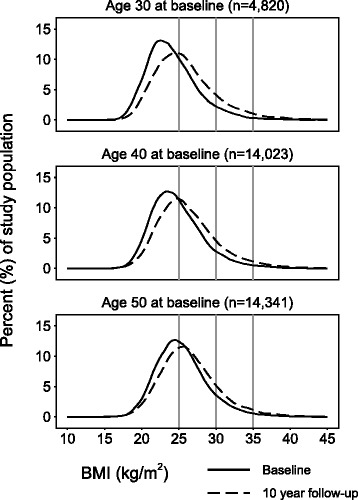
Crude distribution of BMI at baseline (*solid line*) and 10 year follow-up (*dashed line*), by age at baseline. Vӓsterbotten Intervention Programme 1990–2013. Horizontal *grey lines* indicate cut-off points for BMI categories (25.0; 30.0; 35.0 kg/m^2^). Mean (SD) BMI at baseline for ages 30, 40 and 50 was 24.2 (3.6), 24.9 (3.6) and 25.5 (3.6) kg/m^2^, respectively, whereas mean (SD) BMI at 10 year follow-up was 25.9 (4.2), 26.3 (4.2) and 26.5 (4.0) kg/m^2^, respectively. *BMI* Body Mass Index

The mean BMI in the study population was 25.0 (median 24.6, interquartile range (IQR) 22.5-26.9, standard deviation (SD) 3.6) kg/m^2^ at baseline and 26.3 (median 25.8, IQR 23.5–28.4, SD 4.1) kg/m^2^ at 10 year follow-up (Table 1, Fig. 1). At baseline, 55.2% of the participants had a normal BMI (<25.0 kg/m^2^) which fell to 41.2% at 10 year follow-up. In total, 1.1% (*n* = 355) of participants were ever classified as underweight (<18.5 kg/m^2^). Overall, 29.1% of participants maintained their weight (±3%) during follow-up, 56.6% gained weight and 14.2% lost weight.

**Table 1:** Descriptive statistics of study population including all participants with 10 year follow-up, free of prevalent diabetes at baseline and with complete data. Vӓsterbotten Intervention Programme 1990–2013

	BMI at baseline (kg/m^2^)									
	<25.0		25.0–29.9		30.0–34.9		≥35.0		Total	
Total (n, %)^a^	18,332	55.2	11,900	35.9	2,447	7.4	505	1.5	33,184	100.0
Age at baseline, years (n, %)^a^										
30	3,161	65.6	1,345	27.9	257	5.3	57	1.2	4,820	100.0
40	8,137	58.0	4,700	33.5	972	6.9	214	1.5	14,023	100.0
50	7,034	49.0	5,855	40.8	1,218	8.5	234	1.6	14,341	100.0
Sex (n, %)^a^										
Men	7,186	46.5	6,986	45.2	1,147	7.4	140	0.9	15,459	100.0
Women	11,146	62.9	4,914	27.7	1,300	7.3	365	2.1	17,725	100.0
BMI at baseline, kg/m^2^ (mean, SD)	22.5	1.7	26.9	1.3	31.8	1.3	37.9	3.0	25.0	3.6
BMI at 10 year follow-up, kg/m^2^ (mean, SD)	23.9	2.4	28.2	2.5	33.1	3.2	39.0	5.0	26.3	4.1
Change in BMI between baseline and 10 year follow-up, Δkg/m^2^ (mean, SD)	+1.4	1.8	+1.2	2.2	+1.3	2.9	+1.1	4.5	+1.3	2.1
Relative weight change between baseline and 10 year follow-up, as % of baseline weight (mean, SD)	+5.5	8.1	+3.8	8.0	+3.3	9.1	+2.3	11.5	+4.7	8.3
Baseline capillary plasma glucose concentration, mmol/L (mean, SD)										
Fasting	5.2	0.6	5.4	0.6	5.4	0.6	5.5	0.7	5.3	0.6
2-h value	6.3	1.2	6.4	1.3	6.6	1.5	6.9	1.4	6.4	1.3
IGT (n, %)										
At baseline	354	1.9	363	3.1	142	5.8	35	6.9	894	2.7
At 10 year follow-up	900	4.9	966	8.1	304	12.4	70	13.9	2,240	6.8
IFG (n, %)										
At baseline	763	4.2	910	7.6	236	9.6	69	13.7	1,978	6.0
At 10 year follow-up	1,641	9.0	1,414	11.9	338	13.8	81	16.0	3,474	10.5
Year at baseline (n, %)^a^										
1990–1994	7,479	60.3	4,014	32.4	771	6.2	144	1.2	12,408	100.0
1995–1999	8,173	53.2	5,765	37.5	1,177	7.7	248	1.6	15,363	100.0
2000–2003	2,680	49.5	2,121	39.2	499	9.2	113	2.1	5,413	100.0
Education (n, %)										
Primary	6,435	35.1	5,226	43.9	1,163	47.5	260	51.5	13,084	39.4
Any secondary	6,436	35.1	4,037	33.9	838	34.2	170	33.7	11,481	34.6
Any tertiary	5,461	29.8	2,637	22.2	446	18.2	75	14.9	8,619	26.0
Marital status at baseline (n, %)										
Single/Widowed/Divorced	2,951	16.1	1,867	15.7	413	16.9	109	21.6	5,340	16.1
Married/Partner	15,381	83.9	10,033	84.3	2,034	83.1	396	78.4	27,844	83.9
Family history of diabetes (n, %)	2,699	14.7	2,284	19.2	585	23.9	116	23.0	5,684	17.1
Tobacco use at baseline (n, %)	5,839	31.9	4,033	33.9	782	32.0	135	26.7	10,789	32.5
Current smokers	4,017	21.9	2,469	20.7	498	20.4	102	20.2	7,086	21.4
Current snuff users	2,380	13.0	2,094	17.6	383	15.7	45	8.9	4,902	14.8

Percentages calculated in the column except where otherwise indicated

Diabetes, IGT and IFG based on OGTT

*BMI* Body mass index, *IFG* Impaired Fasting Glycaemia, *IGT* Impaired Glucose Tolerance, *OGTT* Oral glucose tolerance test, *SD* Standard deviation

^a^Percentages calculated across the row

Overall, 1087 incident diabetes cases were newly detected at 10 year follow-up, constituting 3.3% of the study population (Table 2). Among the incident diabetes cases, 33.4% had had IGT or IFG at baseline (data not shown). The crude proportion of participants with incident diabetes increased substantially with age, BMI category, and relative weight increase. In addition, men experienced a higher crude diabetes risk than women.

**Table 2:** Cumulative incidence proportion of diabetes at 10 year follow-up by BMI category at baseline. Vӓsterbotten Intervention Programme 1990–2013

	BMI at baseline (kg/m^2^)											
	<25.0			25.0–29.9			30.0–34.9			≥35.0		
	Total (n)	Diabetes events (n)	%	Total (n)	Diabetes events (n)	%	Total (n)	Diabetes events (n)	%	Total (n)	Diabetes events (n)	%
Total	18,332	324	1.8	11,900	500	4.2	2,447	211	8.6	505	52	10.3
Sex												
Men	7,186	154	2.1	6,986	316	4.5	1,147	118	10.3	140	18	12.9
Women	11,146	170	1.5	4,914	184	3.7	1,300	93	7.2	365	34	9.3
Age at baseline												
30	3,161	27	0.9	1,345	20	1.5	257	8	3.1	57	4	7.0
40	8,137	93	1.1	4,700	141	3.0	972	82	8.4	214	23	10.7
50	7,034	204	2.9	5,855	339	5.8	1,218	121	9.9	234	25	10.7
BMI at 10 year follow-up, kg/m^2^												
<25.0	12,665	170	1.3	976	21	2.2	23	1	4.3	2	0	0.0
25.0–29.9	5,481	143	2.6	8,397	314	3.7	317	12	3.8	17	1	5.9
30.0–34.9	173	9	5.2	2,385	144	6.0	1,502	118	7.9	65	4	6.2
≥35.0	13	2	15.4	142	21	14.8	605	80	13.2	421	47	11.2
Weight change between baseline and 10 year follow-up, relative to baseline weight												
Large gain (> +7.0%)	6,745	157	2.3	3,559	199	5.6	772	91	11.8	165	21	12.7
Moderate gain (+3.0 to +7.0%)	4,346	72	1.7	2,632	112	4.3	494	43	8.7	84	10	11.9
Maintenance (±3.0%)	5,111	65	1.3	3,755	139	3.7	679	59	8.7	118	14	11.9
Moderate loss (−3.0 to −7.0%)	1,423	22	1.5	1,135	32	2.8	242	9	3.7	45	5	11.1
Large loss (> −7.0%)	707	8	1.1	819	18	2.2	260	9	3.5	93	2	2.2

*BMI* Body mass index

**Fig. 2:**
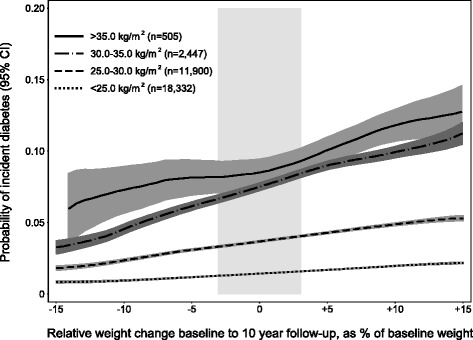
Modelled estimated probability and 95% CI of incident diabetes detected at 10 year follow-up by relative weight change between baseline and 10 year follow-up, and BMI category at baseline (model 4). Vӓsterbotten Intervention Programme 1990–2013. The *grey vertical bar* indicates weight maintenance (±3%). Estimated probabilities adjusted for absolute BMI at baseline (continuous) and the co-variates sex, age at baseline (30, 40 or 50 years), calendar year at baseline (continuous), educational level, marital status, family history of diabetes and tobacco use. *BMI* Body Mass Index, *CI* Confidence Interval

Modelled predicted diabetes risk increased linearly with increase in relative weight change during follow-up in all BMI categories (Fig. 2). Participants with higher BMI at baseline had a higher absolute risk of diabetes but there was no significant difference in relative risk associated with change in relative weight between BMI categories at baseline (*p* = 0.182 for interaction). Overall OR for the linear association with diabetes risk per 1% change in weight was 1.05 (95% CI 1.04, 1.06) (data not shown). There was no evidence of nonlinearity in the association between relative weight change and diabetes risk (*p* = 0.980 for quadratic term). There were no differences in relative risk of diabetes between those aged 40 or 50 years at baseline. With the exception of the highest BMI category, there was no association between BMI change and diabetes risk in the youngest participants who were aged 30 years at baseline (ORs 1.11, 1.14, and 1.08 per unit BMI change, for baseline BMI categories <25.0, 25.0–29.9, and 30.0–34.9, respectively). In the lowest BMI category there was no association between diabetes risk and relative weight reduction (−3.0 to −7.0 or > −7.0%) vs. weight maintenance, whereas there was a substantially increased risk associated with relative weight gain (OR 2.18, 95% CI 1.63, 2.93 for > +7.0% weight change). In the highest BMI category there was no association between relative weight gain and diabetes risk but a strong association with relative weight loss (OR 0.79 (95% CI 0.26, 2.41) for −3.0 to −7.0% weight) (Table 3). Overall results were not affected by excluding participants who were ever underweight (data not shown).

**Table 3:** Association between weight change over 10 years and diabetes risk. Vӓsterbotten Intervention Programme 1990–2013

	BMI at baseline (kg/m^2^)							
	<25.0		25.0–29.9		30.0–34.9		≥35.0	
	OR	95% CI	OR	95% CI	OR	95% CI	OR	95% CI
BMI at 10 year follow-up (model 1)								
<25.0	1.00	Ref	0.63	0.40, 0.98	0.78	0.10, 5.94	-	
25.0–29.9	1.71	1.35, 2.16	1.00	Ref	0.47	0.26, 0.87	0.47	0.06, 3.70
30.0–34.9	4.18	2.07, 8.43	1.62	1.31, 2.01	1.00	Ref	0.53	0.18, 1.54
≥35.0	19.52	4.16, 91.49	5.17	3.14, 8.50	1.87	1.37, 2.56	1.00	Ref
Continuous change in BMI, per unit BMI (1 kg/m^2^) (model 2)								
All	1.24	1.18, 1.31	1.18	1.14, 1.23	1.17	1.11, 1.23	1.13	1.05, 1.21
Age at baseline								
30	1.11	0.94, 1.30	1.08	0.90, 1.28	1.02	0.82, 1.26	1.24	1.00–1.54
40	1.28	1.17, 1.39	1.21	1.14, 1.29	1.16	1.07, 1.25	1.10	1.00–1.22
50	1.26	1.18, 1.35	1.18	1.12, 1.24	1.19	1.10, 1.28	1.09	0.97–1.22
Weight change between baseline and 10 year follow-up, relative to baseline weight (model 3)								
Large gain (> +7.0%)	2.18	1.63, 2.93	1.76	1.40, 2.21	1.65	1.16, 2.35	1.15	0.55, 2.44
Moderate gain (+3.0 to +7.0%)	1.39	0.99, 1.95	1.16	0.90, 1.50	0.99	1.65, 1.61	1.02	0.42, 2.49
Maintenance (±3.0%)	1.00	Ref	1.00	Ref	1.00	Ref	1.00	Ref
Moderate loss (−3.0 to −7.0%)	1.24	0.76, 2.03	0.72	0.48, 1.06	0.38	0.19, 0.79	0.79	0.26, 2.41
Large loss (> −7.0%)	0.87	0.42, 1.84	0.61	0.37, 1.01	0.41	0.20, 0.84	0.12	0.02, 0.54

Estimates adjusted for BMI at baseline (continuous) and the co-variates sex, age at baseline (30, 40 or 50 years), calendar year at baseline (continuous), educational level, marital status, family history of diabetes and tobacco use

*BMI* Body mass index, *CI* Confidence interval, *OR* Odds ratio

PAF results for population shift or maintenance of weight were very similar when defining weight change as absolute BMI change or weight change relative to baseline weight (Table 4). Overall, about 1 in 5 diabetes cases could be prevented if weight in middle age was maintained at the population level; PAF 21.9% (95% CI 15.8, 27.6%) and 22.0% (95% CI 15.5, 28.0%), for maintenance defined as BMI ±1.0 kg/m^2^ or weight ±3.0%, respectively (i). About 2 in 5 cases could be prevented if the population distribution of weight was shifted downward by the equivalent to 5 ± 2% or 1.5 ± 0.5 kg/m^2^; PAF 42.4% (95% CI 24.3, 56.1%) and 38.2% (95% CI 23.4, 50.0%), respectively (ii). As there was no overall benefit with regards to diabetes risk associated with relative weight loss for normal weight participants, we repeated PAF calculations for overweight or obese participants only (PAFs refer to all diabetes cases); PAF 14.3% (95% CI 8.9, 19.1%) for weight maintenance (±1 kg/m^2^) (i), and PAF 23.3% (95% CI 7.6, 35.0%) for population distribution shift of - 1.0 to −2.0 kg/m^2^ in those with ≥25.0 kg/m^2^ at baseline (ii) (data not shown). Overall, 29.6% of the eligible participants at baseline were not followed up (includes deaths and those who moved out of Vӓsterbotten county during the study period). Participants lost to follow-up had higher average baseline BMI (25.6 vs. 25.1 kg/m^2^) and were more likely to have IGT or IFG at baseline (40.0 and 35.6% not followed up, respectively). In addition, we excluded *n* = 2,496 due to missing, incomplete or erroneous data on height/weight or co-variates. There was no difference between the participants with complete and missing data with regards to proportion with diabetes, IGT or IFG at baseline (*p* = 0.799) or at 10 year follow-up (*p* = 0.371), or with regards to average change in BMI between baseline and follow-up (*p* = 0.819, for *n* = 1,423 who had BMI data available but were missing co-variate data).

**Table 4:** Overall association between change in BMI or relative weight and diabetes risk, and population attributable fractions for maintenance or decrease in weight over 10 years. Vӓsterbotten Intervention Programme 1990–2013

	Total (n)	Diabetes events (n)	%	OR	95% CI	PAF		
						Model^a^	(%)	95% CI
Change in BMI between baseline and 10 year follow-up, Δkg/m^2^ (mean, SD)								
Any gain (> +1.0 kg/m^2^)	17,876	701	3.9	1.52	1.32, 1.74	–	–	
Maintenance (±1.0 kg/m^2^)	12,020	317	2.6	1.00	Ref	i	21.9	15.8, 27.6
Moderate loss (−1.0 to −2.0 kg/m^2^)	1,923	45	2.3	0.72	0.52, 0.99	ii	42.4	24.3, 56.1
Large loss (> −2.0 kg/m^2^)	1,365	24	1.8	0.39	0.25, 0.60	iii	68.6	53.5, 78.9
Weight change between baseline and 10 year follow-up, relative to baseline weight								
Any gain (> +3.0%)	18,797	705	3.8	1.51	1.31, 1.75	–	–	
Maintenance (±3.0%)	9,663	277	2.9	1.00	Ref	i	22.0	15.5, 28.0
Moderate loss (−3.0 to −7.0%)	2,845	68	2.4	0.76	0.58, 1.00	ii	38.2	23.4, 50.0
Large loss (> −7.0%)	1,879	37	2.0	0.51	0.36, 0.73	iii	57.5	41.8, 68.9

OR estimates adjusted for BMI at baseline (continuous) and the co-variates sex, age at baseline (30, 40 or 50 years), calendar year at baseline (continuous), educational level, marital status, family history of diabetes and tobacco use

*BMI* Body mass index, *CI* Confidence interval, *OR* Odds ratio, *PAF* Population attributable fraction

^a^The assumed counterfactuals were: weight maintenance vs. any gain (i), moderate weight loss vs. maintenance or any gain (ii), and large weight loss vs. moderate loss or maintenance or any gain (iii)

## Discussion

In this population-based observational cohort study of more than 33,000 participants we have shown that there is a strong association between primary weight maintenance or moderate weight loss and decreased diabetes risk. By estimating PAFs we have also shown that if mean population weight could be shifted downwards by between 1.0 and 2.0 kg/m^2^, 2 in 5 diabetes cases could be prevented. This is equivalent to weight loss of between 3.0 and 6.0 kg for an individual measuring 172.5 cm (the average adult height in Sweden [24]).

Previous trials of lifestyle interventions in high-risk groups (with IGT and high BMI) have achieved average weight loss of 3–6 kg [4, 5], but these intensive health professional-led interventions are costly and could not be delivered at the necessary scale for the whole population at high risk. A more scalable high-risk approach is to use community-based commercial weight loss programmes. A recent study estimated that overweight and obese individuals who completed a 12-week commercial weight loss programme after referral from the NHS, achieved on average −5.6% body weight loss [25]. Based on our data, we can estimate that if all individuals in the population with BMI ≥30.0 kg/m^2^ were enrolled in such a programme, and assuming the effect could be sustained over 10 years, an estimated 8.2% of diabetes cases could be prevented. Considering only about half of participants in trials of behavioural weight loss interventions maintain weight loss for >1 year [26], this is likely greatly overestimated. However, the estimate of 8.2% of diabetes cases prevented if all individuals with BMI ≥30.0 kg/m^2^ were referred to a commercial weight loss programme should be contrasted to *e.g.,* the estimated 21.9% of cases that could be prevented if we achieved population wide weight maintenance (±1 kg/m^2^), clearly illustrating the relative potential of a population-based approach in addition to an intervention targeted to individuals above a defined risk thresh-hold.

We have also shown that as many as 1 in 5 diabetes cases could be prevented if, at a population level, weight was maintained in adulthood. While primary weight maintenance in adulthood is challenging [27], it should be considered in contrast to the even more difficult challenge of weight loss and subsequent secondary weight maintenance [26]. The potential of primary weight maintenance in reducing diabetes risk and burden is an important public health message, and a population-based approach to promote primary weight maintenance (reduce or prevent weight gain in middle age) is desirable as a complement to targeting individuals at high risk.

There is some evidence that identifies potential population level targets associated with weight from modelling studies, *e.g.,* of taxing sugar-sweetened beverages [28], and observational studies, *e.g.,* of the association with the physical environment [29], but the results have shown limited effects. Overall, there is a lack of data available in this field. Two systematic reviews concluded that there is little evidence for community or individual level interventions with the purpose of preventing weight gain in the normal weight population [30, 31], and there has been limited success in preventing weight gain in studies that aimed more broadly to reduce risk factors for cardiovascular disease [31]. While a population-based approach for weight maintenance or moderate weight loss has considerable potential for reducing diabetes burden, single interventions are unlikely to achieve the necessary impact on weight. The barriers and facilitators associated with primary weight maintenance are complex [32], and any effective approach will require changes to policy, environments and health care systems, and incorporate multiple levels and components [33].

Several previous observational population-based studies have examined the association between different levels of weight gain and incident diabetes risk [15], but with a few exceptions [34], there has been less focus on trying to understand the association between moderate weight loss or maintenance on diabetes incidence at a population level. Several previous studies have also tried to estimate PAFs for diabetes associated with the elimination of overweight and obesity with large variation in results [10, 11]. Koh-Banerjee and colleagues estimated the PAF for diabetes incidence associated with weight maintenance between age 21 to the participants age in 1986 (40–75 years) to be 56% in a cohort of 22,171 men [14]. There are several important methodological differences between that study and the present one; for instance, we compared all who maintained their weight to those who gained ≥3.0% or ≥1.0 kg/m^2^ whereas the authors in the previous study had a relatively higher cut-off of ≥7 kg and also included individuals who lost weight in the reference group which would have contributed to a higher estimate of relative risk (RR 3.3 vs equivalent OR 1.52/1.51 in the present study (Table 4)). Ford and colleagues estimated the PAF for diabetes associated with weight maintenance over 7–13 years to be 27% in a cohort of 8545 men and women, using a more similar cut-off of ≥5 kg for weight gain and a similar methodological approach to this study [13]. To our knowledge, the present study is the first to also estimate PAFs for moderate weight loss at a population level.

This study has several strengths, in particular the large sample size, objectively measured exposure and high quality and systematic ascertainment of the outcome of diabetes status based on OGTT. When diabetes is ascertained through self-report or from general practice medical records, there is a danger of differential over diagnosis in groups at high risk, *i.e.,* with a high BMI. However, as all participants in VIP underwent OGTTs, the risk of surveillance (ascertainment) bias of the outcome is minimised. The large population-based sample allowed us to study effects and impact of weight change in individuals at low as well as high risk of diabetes. The fact that we studied intra-individual change in weight as opposed to between-individual difference in weight provides strong support for a causal interpretation of our results.

The primary limitation of this study was that we only had data from two time-points, 10 years apart, and were not able to at present prospectively follow up enough participants for the outcome after the weight change (*e.g.,* to the 20-year follow-up). As one of the symptoms of diabetes is weight loss [17], our results may be somewhat attenuated since diabetes is diagnosed at the same time as the second weight measurement. Similarly, the observational nature of the study means that we cannot distinguish between intentional and unintentional weight loss, which is important with regards to overall mortality risk [35] and thus may be important with regards to diabetes risk as well. To address the potential for bias due to reverse causation we had to exclude participants who self-reported diabetes and we may thus have underestimated the prevalence of diabetes in the study population, and consequently the PAFs are also likely be somewhat underestimated. However, the prevalence of diabetes in the population was comparable to the estimated prevalence in the Swedish population [36]. The VIP protocol did not include measurement of waist circumference during the period of baseline measurements in the present study, and it was thus not possible to study change in visceral adiposity, which may have added value in additional to change in weight in relation to diabetes [37]. About one third of the eligible baseline participants could not be followed up or were excluded due to missing data; participants with IFG or IGT at baseline were more likely not be followed-up and BMI was higher in those lost compared to those who were followed up. As change in weight was associated with baseline BMI and those who were obese on average increased less in weight than normal weight participants, our results may have somewhat overestimated the magnitude of the association between change in weight and diabetes risk. However, since there was no evidence of an interaction between baseline BMI category and change in weight for the association with incident diabetes risk, the resulting bias is likely limited. Mean BMI in the study population (25.0–26.3 kg/m^2^, age 30–60 years) was comparable to the mean BMI in the Swedish population for ages 30–59 years (25.1–26.2 kg/m^2^) [24].

## Conclusions

There is great potential from a public health perspective for diabetes prevention in promoting primary weight maintenance for the whole population, in addition to moderate weight loss in individuals with a BMI ≥25.0 kg/m^2^ and the current approach of targeting interventions to individuals in high risk groups.

## Additional file

Additional file 1: Figure S1. Flowchart describing study population. Vӓsterbotten Intervention Programme 1990-2013. OGTT: Oral glucose tolerance test. (PDF 129 kb)

## Abbreviations

BMI: Body mass index
CI: Confidence interval
IFG: Impaired fasting glycaemia
IGT: Impaired glucose tolerance
kg: Kilogram
m: Metre
OGTT: Oral glucose tolerance test
OR: Odds ratio
PAF: Population attributable fraction
RR: Relative risk
SD: Standard deviation
VIP: Västerbotten intervention programme

## References

[1] Gillies CL, Abrams KR, Lambert PC, Cooper NJ, Sutton AJ, Hsu RT, Khunti K (2007). Pharmacological and lifestyle interventions to prevent or delay type 2 diabetes in people with impaired glucose tolerance: systematic review and meta-analysis. BMJ.

[2] NHS England and Public Health England. National NHS Diabetes Initiative launched in major bid to prevent illness. https://www.gov.uk/government/news/national-nhs-diabetes-initiative-launched-in-major-bid-to-prevent-illness (2015). [Press release published 12/03/2015]. Accessed 18 July 2016.

[3] Schwarz PE, Greaves CJ, Lindstrom J, Yates T, Davies MJ (2012). Nonpharmacological interventions for the prevention of type 2 diabetes mellitus. Nat Rev Endocrinol.

[4] Knowler WC, Barrett-Connor E, Fowler SE, Hamman RF, Lachin JM, Walker EA, Nathan DM, Diabetes Prevention Program Research G (2002). Reduction in the incidence of type 2 diabetes with lifestyle intervention or metformin. N Engl J Med.

[5] Lindstrom J, Louheranta A, Mannelin M, Rastas M, Salminen V, Eriksson J, Uusitupa M, Tuomilehto J, Finnish Diabetes Prevention Study G (2003). The Finnish diabetes prevention study (DPS): lifestyle intervention and 3-year results on diet and physical activity. Diabetes Care.

[6] Abdullah A, Peeters A, de Courten M, Stoelwinder J (2010). The magnitude of association between overweight and obesity and the risk of diabetes: a meta-analysis of prospective cohort studies. Diabetes Res Clin Pract.

[7] Hartemink N, Boshuizen HC, Nagelkerke NJ, Jacobs MA, van Houwelingen HC (2006). Combining risk estimates from observational studies with different exposure cutpoints: a meta-analysis on body mass index and diabetes type 2. Am J Epidemiol.

[8] Rose G (1985). Sick individuals and sick populations. Int J Epidemiol.

[9] Flegal KM, Panagiotou OA, Graubard BI (2015). Estimating population attributable fractions to quantify the health burden of obesity. Ann Epidemiol.

[10] Wilson PW, D’Agostino RB, Sullivan L, Parise H, Kannel WB (2002). Overweight and obesity as determinants of cardiovascular risk: the Framingham experience. Arch Intern Med.

[11] Laaksonen MA, Knekt P, Rissanen H, Harkanen T, Virtala E, Marniemi J, Aromaa A, Heliovaara M, Reunanen A (2010). The relative importance of modifiable potential risk factors of type 2 diabetes: a meta-analysis of two cohorts. Eur J Epidemiol.

[12] Fildes A, Charlton J, Rudisill C, Littlejohns P, Prevost AT, Gulliford MC. Probability of an Obese Person Attaining Normal Body Weight: Cohort Study Using Electronic Health Records. Am J Public Health. 2015;105(9):e54–9. 10.2105/AJPH.2015.302773 PMC4539812 26180980

[13] Ford ES, Williamson DF, Liu S (1997). Weight change and diabetes incidence: findings from a national cohort of US adults. Am J Epidemiol.

[14] Koh-Banerjee P, Wang Y, Hu FB, Spiegelman D, Willett WC, Rimm EB (2004). Changes in body weight and body fat distribution as risk factors for clinical diabetes in US men. Am J Epidemiol.

[15] Kodama S, Horikawa C, Fujihara K, Yoshizawa S, Yachi Y, Tanaka S, Ohara N, Matsunaga S, Yamada T, Hanyu O (2014). Quantitative relationship between body weight gain in adulthood and incident type 2 diabetes: a meta-analysis. Obes Rev.

[16] Norberg M, Wall S, Boman K, Weinehall L. The Västerbotten Intervention Programme: background, design and implications. Glob Health Action. 2010;3. 10.3402/gha.v3i0.4643 PMC2844807 20339479

[17] de Fine Olivarius N, Siersma VD, Koster-Rasmussen R, Heitmann BL, Waldorff FB (2015). Weight changes following the diagnosis of type 2 diabetes: the impact of recent and past weight history before diagnosis. results from the Danish Diabetes Care in General Practice (DCGP) study. PLoS One.

[18] World Health Organization (1999). Definition, diagnosis and classification of diabetes mellitus and its complications. Report of a WHO Consultation. Part 1: Diagnosis and Classification of Diabetes Mellitus.

[19] Alberti KG, Zimmet PZ (1998). Definition, diagnosis and classification of diabetes mellitus and its complications. Part 1: diagnosis and classification of diabetes mellitus provisional report of a WHO consultation. Diabet Med.

[20] Sorkin JD, Muller DC, Andres R (1999). Longitudinal change in the heights of men and women: consequential effects on body mass index. Epidemiol Rev.

[21] Stevens J, Truesdale KP, McClain JE, Cai J (2006). The definition of weight maintenance. Int J Obes.

[22] Rockhill B, Weinberg CR, Newman B (1998). Population attributable fraction estimation for established breast cancer risk factors: considering the issues of high prevalence and unmodifiability. Am J Epidemiol.

[23] Zhang J, Yu KF (1998). What’s the relative risk? A method of correcting the odds ratio in cohort studies of common outcomes. JAMA.

[24] Statistics Sweden. The Swedish Living Conditions Survey. http://www.scb.se/le0101. [In Swedish, published 20/09/2013]. Accessed 11 Jan 2017.

[25] Ahern AL, Olson AD, Aston LM, Jebb SA (2011). Weight Watchers on prescription: an observational study of weight change among adults referred to Weight Watchers by the NHS. BMC Public Health.

[26] Barte JC, ter Bogt NC, Bogers RP, Teixeira PJ, Blissmer B, Mori TA, Bemelmans WJ (2010). Maintenance of weight loss after lifestyle interventions for overweight and obesity, a systematic review. Obes Rev.

[27] Lindvall K, Larsson C, Weinehall L, Emmelin M (2010). Weight maintenance as a tight rope walk - a Grounded Theory study. BMC Public Health.

[28] Briggs AD, Mytton OT, Kehlbacher A, Tiffin R, Rayner M, Scarborough P (2013). Overall and income specific effect on prevalence of overweight and obesity of 20% sugar sweetened drink tax in UK: econometric and comparative risk assessment modelling study. BMJ.

[29] Mackenbach JD, Rutter H, Compernolle S, Glonti K, Oppert JM, Charreire H, De Bourdeaudhuij I, Brug J, Nijpels G, Lakerveld J (2014). Obesogenic environments: a systematic review of the association between the physical environment and adult weight status, the SPOTLIGHT project. BMC Public Health.

[30] Peirson L, Douketis J, Ciliska D, Fitzpatrick-Lewis D, Ali MU, Raina P (2014). Prevention of overweight and obesity in adult populations: a systematic review. CMAJ Open.

[31] Reeder BA, Katzmarzyk PT (2006). Canadian task force on preventive health care. Prevention of weight gain and obesity in adults: a systematic review.

[32] Nafziger AN, Lindvall K, Norberg M, Stenlund H, Wall S, Jenkins PL, Pearson TA, Weinehall L (2007). Who is maintaining weight in a middle-aged population in Sweden? A longitudinal analysis over 10 years. BMC Public Health.

[33] Swinburn BA, Sacks G, Hall KD, McPherson K, Finegood DT, Moodie ML, Gortmaker SL (2011). The global obesity pandemic: shaped by global drivers and local environments. Lancet.

[34] Mishra GD, Carrigan G, Brown WJ, Barnett AG, Dobson AJ (2007). Short-term weight change and the incidence of diabetes in midlife: results from the Australian Longitudinal Study on Women’s Health. Diabetes Care.

[35] Harrington M, Gibson S, Cottrell RC (2009). A review and meta-analysis of the effect of weight loss on all-cause mortality risk. Nutr Res Rev.

[36] Jansson SP, Fall K, Brus O, Magnuson A, Wandell P, Ostgren CJ, Rolandsson O (2015). Prevalence and incidence of diabetes mellitus: a nationwide population-based pharmaco-epidemiological study in Sweden. Diabet Med.

[37] The InterAct Consortium (2012). Long-term risk of incident type 2 diabetes and measures of overall and regional obesity: the EPIC-InterAct case-cohort study. PLoS Med.

