# Supplementary material for: Impact of weight maintenance and loss on diabetes risk and burden: a population-based study in 33,184 participants
Source: BMC Public Health. 2017 Feb 6;17:170. doi: 10.1186/s12889-017-4081-6 (PMC5294882; doi:10.1186/s12889-017-4081-6)
Supplement: Additional file 1: Figure S1. — Flowchart describing study population. Vӓsterbotten Intervention Programme 1990-2013. OGTT: Oral glucose tolerance test. (PDF 129 kb) [file 12889_2017_4081_MOESM1_ESM.pdf]

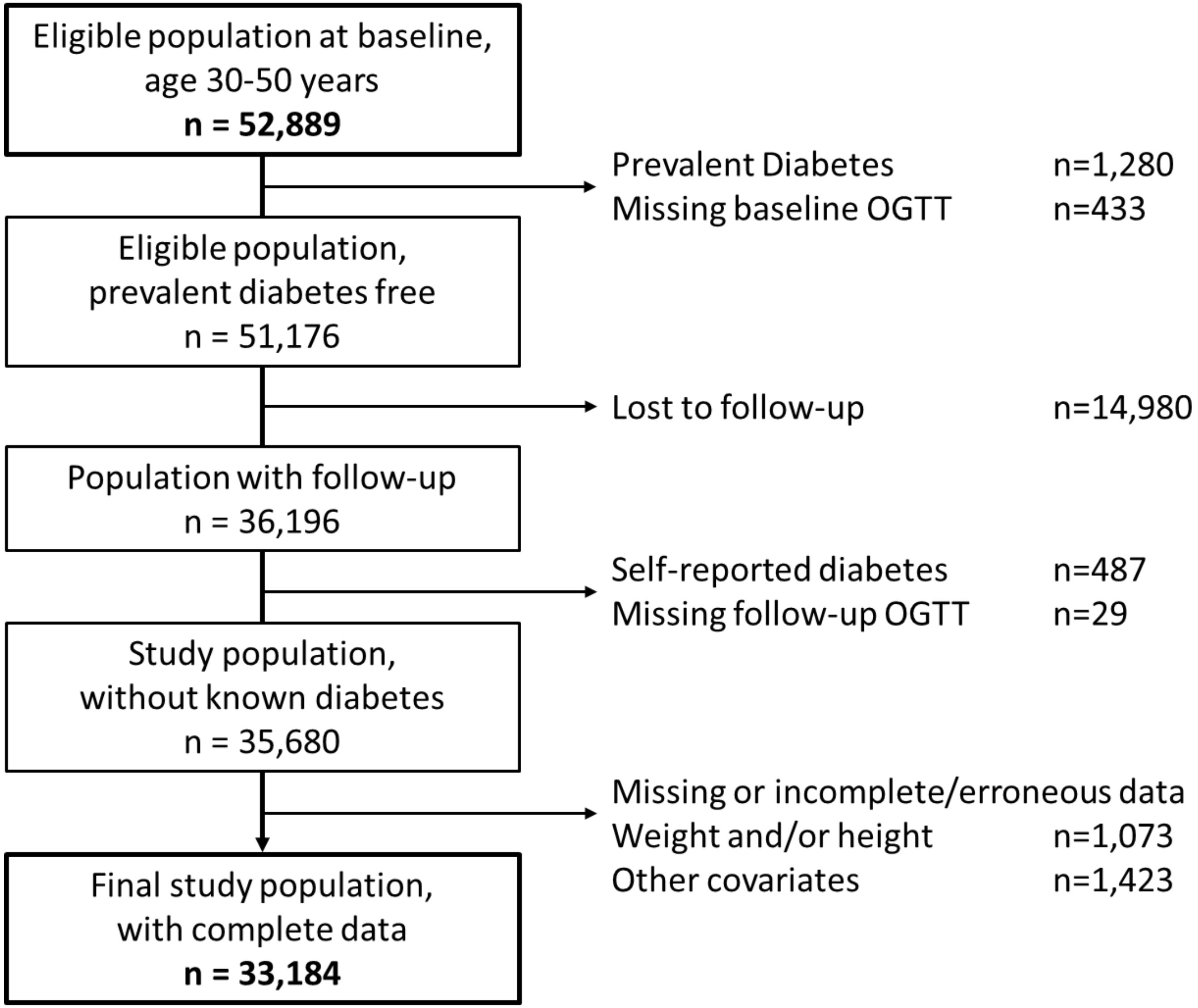

Supplemental figure: Flowchart describing study population. Västerbotten Intervention Programme 1990-2013.
OGTT: Oral glucose tolerance test.
